# Evaluation of the relationship between cognitive impairment and suboptimal health status in a northern Chinese population: a cross-sectional study

**DOI:** 10.7189/jogh.10.010804

**Published:** 2020-06

**Authors:** Guoyong Ding, Xuan Zhao, Youxin Wang, Daiyu Song, Dongzhen Chen, Yang Deng, Weijia Xing, Hualei Dong, Yong Zhou, Dong Li, Haifeng Hou

**Affiliations:** 1 School of Public Health, Shandong First Medical University & Shandong Academy of Medical Sciences, Taian, Shandong Province, China; 2 Beijing Key Laboratory of Clinical Epidemiology, School of Public Health, Capital Medical University, Beijing, China; 3 Taishan Hospital of Shandong Province, Taian, Shandong Province, China; 4 Eye Hospital, Wenzhou Medical University, Wenzhou, Zhejiang Province, China; * Equal authorship.

## Abstract

**Background:**

Suboptimal health status (SHS) is an intermediate health status between ideal health and illness. As a determinant of cardiovascular disease and stroke, SHS is hypothesized to be associated with the development of cognitive impairment and dementia. This study aimed to investigate whether individuals with SHS have poor cognitive ability based on a community-based cohort in northern Chinese population.

**Methods:**

3524 participants who were enrolled in *Jidong* cohort 2015 in Tangshan City were investigated in this study. Cognitive function was measured with the Mini-Mental State Examination (MMSE). SHS level was evaluated using a self-reporting Suboptimal Health Status Questionnaire-25 (SHSQ-25). The relationship between SHS and cognitive function was analyzed with logistic regression analysis, by which odds ratio (OR) and 95% confidence interval (CI) were calculated.

**Results:**

The prevalence of cognitive impairment was 3.4% (121/3524) in our study, with the prevalence rates of 1.9% (34/1750) among men and 4.9% (87/1774) in women. The medians of total score of MMSE were 28 (interquartile range (IQR) = 27-29) in the SHS group, and 29 (IQR = 27-30) in the ideal health group. Logistic regression analysis showed that SHS was significantly correlated with cognitive impairment (adjusted OR = 2.936, 95% CI = 1.428-6.033). With regard to gender, the OR was 5.067 (95% CI = 1.346-19.068) in men, which was higher than that in women (OR = 2.324, 95% CI = 1.130-4.779).

**Conclusions:**

SHS might be a risk factor for cognitive function in northern Chinese population. Early screening of SHS individuals, as well as urgent treatment of SHS might contribute to the prevention of cognitive impairment.

Cognitive impairment is the intermediate stage between the intact cognitive functioning and early clinical dementia [1]. The incidences of cognitive impairment and dementia have been increasing worldwide [2]. And the number of new diagnosed dementia is 7.7 million each year, and will be 75.6 million by 2030[2]. In the Asia-Pacific region, the number of dementia patients is expected to increase from 27 million in 2015 to 70 million in 2050 [3]. With the acceleration of ageing of the population process, cognitive impairment has become an increasing challenge for health care systems in China, where the corresponding caregivers are urgently needed [4]. As no medications can effectively reverse the progression of dementia, identifying the modifiable risk factors, as well as lessening the exposure to these factors is crucial for the reduction of the burdens.

Suboptimal health status (SHS) is an intermediate health status between ideal health and illness [5]. As a reversible subclinical stage, SHS was described as perceptible discomfort symptoms, such as health complaint, self-reported weakness, chronic fatigue, non-specific pain and psychological symptom [6]. Advances have been acquired for measurement and evaluation of SHS in these years. Suboptimal Health Status Questionnaire-25 (SHSQ-25) is the most widely used scale for measurement of SHS [7,8], which has been used in Africans, Asians and Caucasians [5]. Accumulating evidences indicated the associations between SHS and a wide range of diseases including mental disorders [9,10], psychosocial stress [11], obsessive-compulsive symptom and depression symptom [12], hypercholesterolemia [13], overweight and obesity [14], stroke [15,16], type II diabetes and cardiovascular disease [17]. All these diseases are related to the development of dementia. Therefore, we conducted this study to estimate the prevalence rates of cognitive impairment among a northern Chinese population, and investigated whether cognitive impairment was associated with SHS.

## METHODS

### Study design and participants

This study was approved by the Ethics Committee of Shandong First Medical University and the Ethic Committee of Workers' Hospital of *Jidong* Oilfield of Petro China (No. 01-2013). The participants in this study were recruited from *Jidong* cognitive impairment cohort study (CICS) in Tangshan City in 2015. CICS is a community-based, prospective, long-term observational cohort study to evaluate potential risk factors and prognosis of cognitive impairment [18]. Tangshan is located in the central part of the Bohai Rim, adjacent to Beijing and Tianjin.

**Figure 1:**
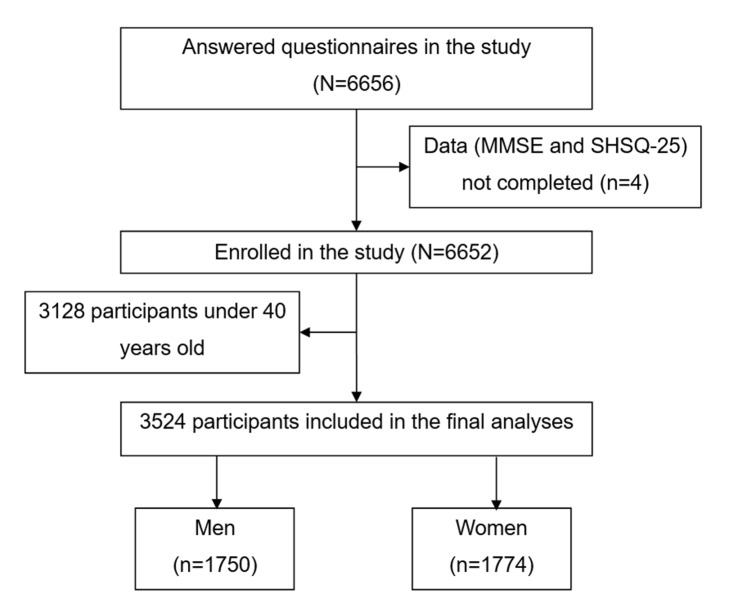
Selection procedures of the study. MMSE – Mini-Mental State Examination; SHSQ-25 – Suboptimal Health Status Questionnaire-25.

The inclusion criteria of the study were: 1) age >40 years; 2) local Han population; 3) complete basic information available; 4) signed informed consent. A total of 6656 people agreed to participate in the questionnaire survey, among which 3128 individuals were under 40 years old, three did not complete the Mini-Mental State Examination (MMSE) questionnaire and one did not complete the SHSQ-25 questionnaire. Finally, we included 3524 participants in this study (**Figure 1**).

### Anthropometric variables and clinical data

Data were collected via a single visit by experienced physicians and trained nurses, where a standard questionnaire was used by a face-to-face interview. We collected the following information of the participants: 1) personal information (gender, age, educational level, living status, body mass index (the ratio of weight, kg / height squared, m^2^), per-capita living space, number of family members, family environment and family hygiene), 2) disease history (hypertension, diabetes, hyperlipemia, coronary heart disease (CHD), fatty liver, autoimmune disease, respiratory disease, chronic gastritis, gastric ulcer and duodenal ulcer), 3) lifestyle (smoking, drinking and staying up late), and 4) dietary habits.

### Cognitive impairment evaluation

Cognitive function was measured with the MMSE [19], which consists of 30 items that evaluate five cognitive dimensions: 1) orientation to time and place (10 points), 2) word registration (3 points), 3) attention and calculation (5 points), 4) recall (3 points) and 5) language (containing visual construction, 9 points). The scores of MMSE range from 0 to 30, the higher scores indicate better cognitive function. The MMSE scale has a sensitivity above 88.3% and a specificity of 87% with a cut-off point of 24 for detecting cognitive impairment in patients with neurodegeneration [20,21]. In this study, cognitive impairment was defined as: a score ≤17 for illiterates; a score ≤20 for primary school graduates (≥6 years of education); and a score ≤24 for junior school graduates or above (≥9 years of education) [22,23].

### SHS evaluation

The condition of SHS was measure by the questionnaire SHSQ-25 (Table S1 in the **Online Supplementary Document**). The SHSQ-25 includes 25 items on five dimensions: 1) fatigue, 2) cardiovascular system, 3) digestive tract, 4) immune system and 5) mental health [12,17]. According to how often respondents experienced symptoms in the previous three months, each item was presented in a specific form on a Likert scale on a five-point scale. The raw scores of 1-5 on the questionnaire were recorded as 0-4. The total score of SHSQ-25 of each subject was obtained by summing the scores of the 25 items. The conditions of participants were stratified into two classifications: ideal health (with summed score <35) and suboptimal health status (with summed score ≥35) [17,19]. The SHSQ-25 has good individual internal consistency with the Cronbach’s α coefficient was 0.91 [5]. Studies have showed that the SHSQ-25 is a reliable and valid instrument for measuring sub-health status in Chinese population [24,25].

### Statistical analysis

All the statistical analyses were performed by SPSS (version 25.0, IBM, New York, USA). Qualitative data were presented as a rate or percentage. The normality of continuous variables was conducted by using the Shapiro-Wilk test. Continuous variables of normality distributions were expressed as mean ± standard deviation (SD). Median and interquartile range (IQR) were used for non-normally distributed data. The diﬀerences between the healthy group and SHS group were tested by *t* test for normally distributed data and Wilcoxon rank sum test for non-normally distributed or graded variables, or chi-square (χ^2^) test (discrete variables). The Cochran Armitage trend test was adopted to analyze the linear trend between cognitive impairment and educational level. Multivariate logistic regression was used to examine the relationship between cognitive impairment and SHS, where odds ratio (OR) and 95% confidence interval (CI) were calculated. *P* value of less than 0.05 was considered statistically significant.

## RESULTS

### Characteristics of participants

Among 3524 participants (mean age 54.57 ± 8.74 years) included in this study, 1750 (49.7%) were men (mean age 54.88 ± 8.98 years) and 1774 (50.3%) were women (mean age 54.27 ± 8.48 years). All the participants were classified into two groups on the basis of the SHSQ-25 scores: healthy group (n = 3381) and SHS group (n = 143). The characteristics of the participants in each group are presented in **Table 1**. The differences of SHS prevalence were not significant between age-groups. In addition, education level, living status, BMI, per-capita living space, family environment, and family hygiene classifications were not related to SHS prevalence. While, the prevalence of SHS among women was significantly higher than that among men (*P* < 0.001). And the prevalence among the participants lived alone was 7.2%, which was significantly higher than participants lived with his/her families (*P* = 0.020).

**Table 1:** Demographic characteristics of study participants among healthy and suboptimal health status groups.

Characteristics	Total	Healthy	SHS	χ^2^/t	*P*
**Age** (years)*	54.57 ± 8.74	54.52 ± 8.75	55.72 ± 8.30	1.607	0.108
**BMI** (kg/m^2^)*	24.94 ± 3.29	24.96 ± 3.28	24.46 ± 3.62	1.751	0.080
**Gender:**					
Male (n, %)	1750	1707 (97.5)	43 (2.5)	22.880	<0.001
Female (n, %)	1774	1647 (94.5)	100 (5.6)		
**Education:**					
Illiterate (n, %)	84	78 (92.9)	6 (7.1)	6.802	0.078
Primary (n, %)	296	283 (95.6)	13 (4.4)		
Middle (n, %)	1874	1788 (95.4)	86 (4.6)		
College (n, %)	1251	1213 (97.0)	38 (3.0)		
**Living alone** (n, %)	98	93 (94.9)	5 (5.1)	0.323	0.570
**Per-capita living space (**m^2^**):**					
≤30 (n, %)	792	761 (96.1)	31 (3.9)	0.688	0.709
30-59 (n, %)	1430	1376 (96.2)	54 (3.8)		
≥60 (n, %)	1300	1243 (95.6)	57 (4.4)		
**N. of family members:**					
one (n, %)	138	128 (92.8)	10 (7.2)	7.862	0.020
two - four (n, %)	3198	3067 (95.9)	131 (4.1)		
Five or more (n, %)	186	184 (98.9)	2 (1.1)		
**Family hygiene:**					
Very Good (n, %)	2397	2306 (96.2)	91 (3.8)	1.188	0.552
Good (n, %)	1110	1060 (95.5)	50 (4.5)		
Poor (n, %)	16	15 (93.8)	1 (6.3)		

SHS – suboptimal health status, BMI – body mass index

*Data were expressed as mean and standard deviation.

### The prevalence of cognitive impairment

**Figure 2:**
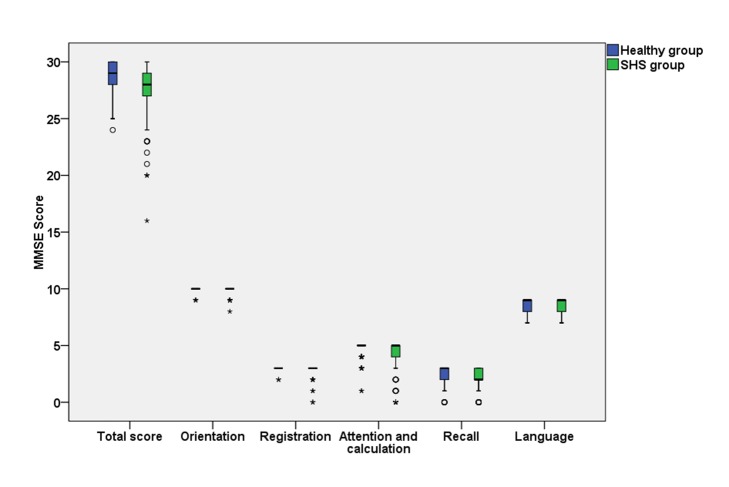
Box plot of Mini-Mental State Examination scores in the healthy and suboptimal healthy status groups. The data were expressed as minimum, P_25_, median, P_75_, maximum, outlier and extreme. SHS – suboptimal healthy status.

The MMSE score in the healthy and SHS groups were shown in **Figure 2** and Table S2 in the **Online Supplementary Document**. The median of total score of MMSE was 29 (IQR: 27-30) in the healthy group and 28 (IQR: 27-29) in the SHS group. Compared with health participants, SHS individuals had lower cognitive function in overall level (*P* = 0.001), word registration domain (*P* = 0.010), and attention and calculation domain (*P* = 0.001).

The prevalence of cognitive impairment was 3.4% (121/3524) in this study, with 1.9% (34/1750) among men and 4.9% (87/1774) among women. As shown in **Table 2**, the prevalence rates of cognitive impairment had a significant difference between age groups (*P* < 0.001). The prevalence of cognitive impairment was relatively high in people over the age of 61. The prevalence rate of cognitive impairment was highest in the participants with illiterate (40.5%). The trend that the prevalence of cognitive impairment decreased with the increase of education level was also observed in both men and women groups (Total: *P* < 0.001; Men: *P* < 0.001; Women: *P* < 0.001). The prevalence of cognitive impairment for lived alone individuals (9.2%) was higher than that of not lived alone individuals (3.3%, *P* = 0.004). For the classifications of per-capita living space, the prevalence rates of cognitive impairment were statistically different between groups (Table S3 in the **Online Supplementary Document**, *P* = 0.014). No significant differences were found for subgroup analyses on BMI, number of family members, family environment and family hygiene (Table S3 in the **Online Supplementary Document**).

**Table 2:** Prevalence rates of cognitive impairment in the subgroups of demographics*

Subgroups	No.	Cases	Male cases	Female cases
**Age (years):**				
41-45 (n, %)	655	5 (0.8)^a^	2 (0.6)^a^	3 (0.9)^a^
46-50 (n, %)	719	7 (1.0)^a^	2 (0.6)^a^	5 (1.4)^a^
51-55 (n, %)	541	9 (1.7)^a,b^	2 (0.8)^a^	7 (2.5)^a,b^
56-60 (n, %)	517	22 (4.3)^b,c^	5 (2.2)^a,b^	17 (5.9)^b,c^
61-65 (n, %)	695	41 (5.9)^c,d^	8 (2.2)^a,b^	33 (9.9)^c^
≥66 (n, %)	397	37 (9.3)^d^	15 (6.7)^b^	22 (12.6)^c^
*P-*value		<0.001	<0.001	<0.001
**Education:†**				
Illiterate (n, %)	84	34 (40.5)^a^	5 (26.3)^a^	29 (44.6)^a^
Primary (n, %)	296	40 (13.5)^b^	11 (9.6)^a^	29 (16.0)^b^
Middle (n, %)	1874	40 (2.1)^c^	15 (1.7)^b^	25 (2.5)^c^
College (n, %)	1251	5 (0.4)^d^	2 (0.3)^c^	3 (0.6)^d^
*P*-value		<0.001	<0.001	<0.001
**Living status:**				
Living alone (n, %)	98	9 (9.2)	0 (0)	9 (10.5)
Not living alone (n, %)	3231	107 (3.3)	30 (1.9)	77 (4.8)
*P value*		0.004	0.391	0.001
**BMI (kg/m^2^):**				
<18.5 (n, %)	120	3 (2.5)	0 (0)	3 (9.1)
18.5-23.9 (n, %)	1310	38 (2.9)	7 (1.5)	31 (3.7)
24-27 (n, %)	1444	52 (3.6)	15 (1.8)	37 (6.1)
≥28 (n, %)	581	25 (4.3)	10 (3.0)	15 (6.0)
*P*-value		0.277	0.442	0.099

*Same letters marked in any two subgroups indicate no statistically significant differences. Completely different letters in any two subgroups indicate statistically significant differences.

†Cochran Armitage trend test: Total, χ^2^ = 300.678, *P* < 0.001; Men, χ^2^ = 66.042, *P* < 0.001; Women, χ^2^ = 204.453, *P* < 0.001.

### The relationship of cognitive impairment with lifestyles and dietary habits

As shown in **Table 3** and Table S4 in the **Online Supplementary Document**, the prevalence of cognitive impairment was significantly associated with smoking habit (*P* = 0.018), drinking (*P* < 0.001), fried food (*P* = 0.001) and spicy food intakes (*P* = 0.042), and staying up late (*P* = 0.025). With regard to gender, cognitive impairment was significantly associated with fried food (*P* < 0.001), spicy food (*P* = 0.013), pickled food (*P* < 0.001) and bacon intakes (*P* < 0.001) among men. While only staying up late was significantly associated with cognitive impairment among women (*P* = 0.021).

**Table 3:** Prevalence rates of cognitive impairment in behavior-related subgroups.

Behavioral factors	No.	Cases	Male cases	Female cases
**Smoke status:**				
Non-smokers (n, %)	2500	97 (3.9)^a^	14 (1.8)	83 (4.8)
Smokers (n, %)	844	16 (1.9)^b^	13 (1.6)	3 (8.1)
Former smokers (n, %)	180	8 (4.4)^a,b^	7 (4.0)	1 (25.0)
*P*-value		0.018	0.114	0.115
**Daily smoking amount:**				
0 cigarettes per day (n, %)	2667	106 (4.0)^a^	22 (2.3)	84 (4.9)
1-10 cigarettes per day (n, %)	315	7 (2.2)^a,b^	4 (1.4)	3 (11.1)
11-20 cigarettes per day (n, %)	399	6 (1.5)^b^	6 (1.6)	0 (0)
≥21 cigarettes per day (n, %)	143	2 (1.4)^a,b^	2 (1.4)	0 (0)
*P*-value		0.018	0.659	0.305
**Passive smoking:**				
Yes (n, %)	1061	37 (3.5)	10 (1.9)	27 (5.1)
No (n, %)	2463	84 (3.4)	24 (2.0)	60 (4.8)
*P*-value		0.909	0.928	0.846
Drinking:				
Yes (n, %)	1106	16 (1.4)	14 (1.4)	2 (1.8)
No (n, %)	2343	102 (4.4)	17 (2.4)	85 (5.2)
*P-*value		<0.001	0.141	0.109

*Same letters marked in any two subgroups indicate no statistically significant differences. Completely different letters in any two subgroups indicate statistically significant differences.

### The relationship between cognitive impairment and disease history

**Figure 3:**
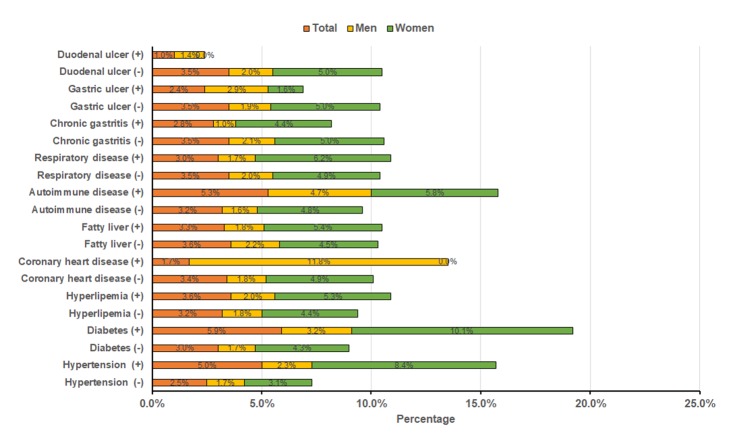
Percentages of cognitive impairment among participants with a history of disease.

**Figure 3** shows percentages of cognitive impairment among participants with or without a history of disease. The percentages of cognitive impairment among total participants with hypertension (5.0% vs 2.5%), diabetes (5.9% vs 3.0%) and autoimmune (5.3% vs 3.2%) were significantly higher than that among participants without these diseases (all *P* < 0.05). In male participants, the percentages of cognitive impairment among participants with CHD (11.8% vs 1.8%) and autoimmune (4.7% vs 1.6%) than that among participants without these diseases (all *P* < 0.05). Chi-square tests showed that cognitive impairment was significantly correlated with hypertension (*P* < 0.001) and diabetes (*P* < 0.001) among female participants.

### Multivariate logistic regression analysis on determinants for cognitive impairment

**Figure 4:**
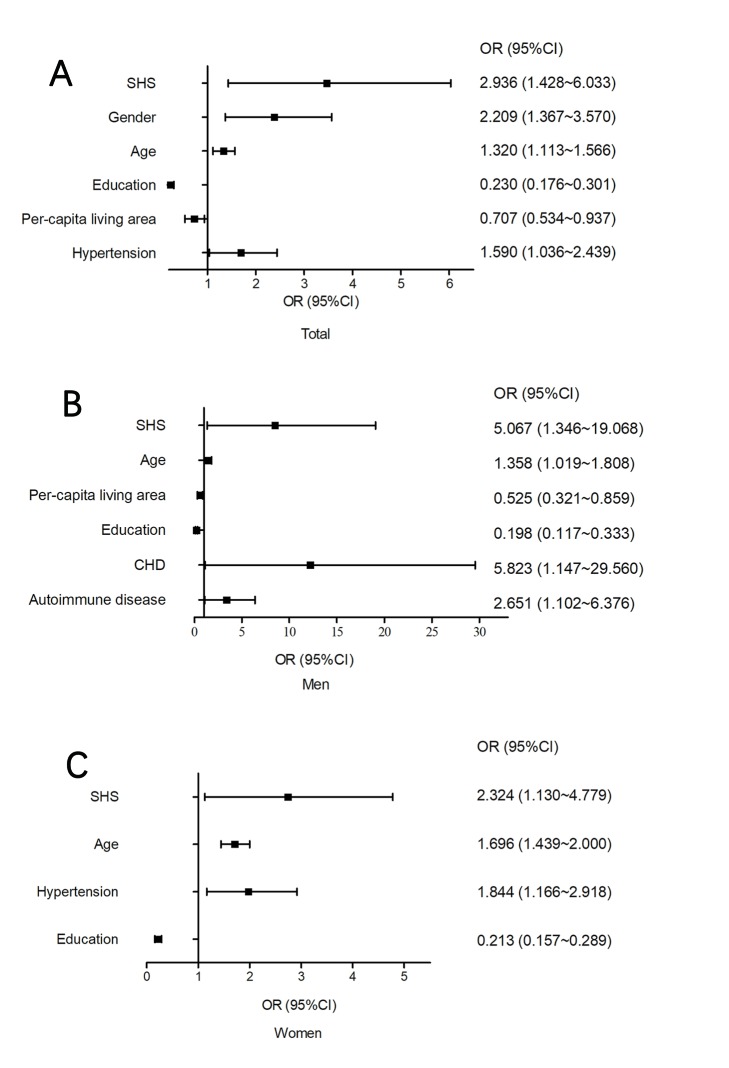
Multivariate logistic regression analysis for cognitive impairment. (**A**) Among total participants; (**B**) among men participants; (**C**) among women participants. SHS – suboptimal health status; CHD – coronary heart disease.

As shown in **Figure 4**, Panel A, and Figure S1 in the **Online Supplementary Document**, SHS was significantly correlated with an increased risk of cognitive impairment (OR = 2.936, 95% CI = 1.428-6.033) after adjusting for other factors (gender, age, education, per-capita living area, a history of disease). In addition, gender (*P* = 0.001), age (*P* = 0.001) and hypertension (*P* = 0.034) were positively correlated with cognitive impairment, while education and per-capita living space (*P* = 0.016) were negatively correlated with cognitive impairment. Among the male participants, age (*P* = 0.037), per-capita living space (*P* = 0.010), education level (*P* < 0.001), CHD (*P* = 0.034) and autoimmune disease (*P* = 0.029) were correlated with cognitive impairment (**Figure 4**, Panel B). While, cognitive impairment was significantly correlated with age (*P* < 0.001), hypertension (*P* = 0.009) and education level (*P* < 0.001) among women (**Figure 4****,** Panel C).

## DISCUSSION

This study has, for the first time, investigated the relationship between SHS and cognitive impairment in middle-aged and older Chinese adults. Our findings indicate that SHS participants tend to have lower cognitive function and SHS might be a risk factor for cognitive impairment. This finding was validated in multivariate logistic regression analysis, which also showed that SHS was significant determinants of cognitive impairment in addition to gender, age, education level, per-capita living space and hypertension.

We also analyzed the prevalence rates of cognitive impairment in subgroups of demographics. Our finding on gender difference was consistent with a published study on a Chinese elderly population, where the prevalence of women (32.9%) was twice as high as that among men (15.7%) [26]. These results indicate women experience higher risk for this condition than men [27,28]. We also observed the higher prevalence of cognitive impairment in elder participants, validating that the trends of risk go up with increasing age among both men and women [29,30]. With regard to the effect of education levels, our study supported that individuals with lower attained education suffered more prevalence of cognitive impairment [31,32]. In terms of living status, we found the occurrence of cognitive impairment might be aggravated by living alone [31]. Interestingly, it is found that people lived in smaller house (measured by the per-capita living space) were exposed to higher risk of cognitive impairment. As is known, living space is related to economic condition or income of residents, which is one of important determinants of cognitive impairment [31,33,34].

Behavior-related factors (eg, staying up late) were also observed to be associated with the prevalence of cognitive impairment in this study. It has been found that sleep disorder is not only an important risk factors for cognitive impairment, but also a symptom of neurodegenerative process [35-38]. Among male participants, dietary factors (eg, fried food, pickled food, spicy food and bacon intakes) were strongly associated with cognitive impairment. However, some other previously acknowledged risk factors such as alcohol drinking [39], smoking [40] were found to be non-significant in this study, which might be attributed to the fact that most of participants with cognitive impairment were women (71.9%). Furthermore, the percentage of cognitive impairment in our study was higher among the participants with hypertension, diabetes and CHD, which has been validated in previous researches [41-43]. These diseases commonly share similar vascular-related risk factors with cognitive impairment, in addition to their underlining contributions to neurodegeneration.

This study identified significant relationship of SHS with cognitive impairment, where oxidative stress might play an important role [44]. Oxidative stress increases the release of a variety of growth factors and molecules, which up-regulate vascular permeability, protein exudation and demyelination of axons, and result in potential disfunction of brain tissue [45,46]. In the SHS stage, individuals are exposed to aberrant oxidative stress, which leads to vascular lesions and increases the risk of cardiovascular diseases [47,48]. In addition, other researchers found vascular biochemical changes and structural changes in Alzheimer disease (AD) patients [49]. The destruction of blood-brain barrier and neurovascular units is an important pathogenesis of cognitive impairment [50]. Vascular lesion is one of the main causes of dementia in the elderly, suggesting that lesions have a role in cognitive impairment [51-53]. Therefore, SHS might be associated with cognitive impairment by peroxidation stress and vascular lesions.

Dementia have been a common public health burden, which threatens 47 million people worldwide, and this number is expected to increase to 131 million by 2050 [54]. China has the largest number of patients who suffer from dementia that imposes a huge challenge for health care system that has been facing the burden of aging of population [55]. Identification of persons at high risk of dementia and early diagnosis of cognitive impairment, as well as clinical management and care, are still urgently required in China. As far as we know, this is the first study to elucidate the relationship between SHS and cognitive impairment of in Chinese population.

Some limitations of our study should be acknowledged. First, since this study is a cross-sectional study, it is difficult to infer a causality relationship of SHS with cognitive impairment, and the pathophysiological mechanism could not be depicted. Second, MMSE is a simplified scale for cognitive function measurement, and cannot represent clinical evaluation of cognitive impairment or dementia, the accuracy of the results is, more or less, influenced.

In a conclusion, SHS might be a risk factors for cognitive function in a northern Chinese population. Cohort studies are still needed to assess the causal relationship between SHS and cognitive impairment. Early screening of SHS individuals, as well as urgent treatment of SHS is needed to effectively promote the prevention of cognitive impairment.

## Additional material

Online Supplementary Document
