# Supplementary material for: Evaluation of the relationship between cognitive impairment and suboptimal health status in a northern Chinese population: a cross-sectional study
Source: J Glob Health. 2020 Feb 20;10(1):010804. doi: 10.7189/jogh.10.010804 (PMC7101211; doi:10.7189/jogh.10.010804)
Supplement: Online Supplementary Document [file jogh-10-010804-s001.pdf]

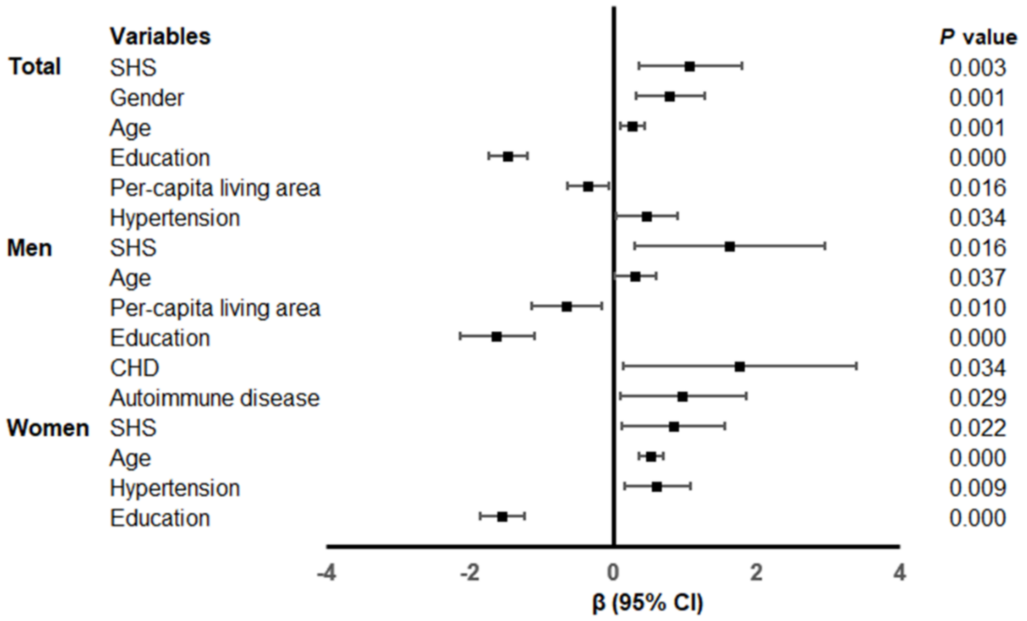

Figure S1 Regression coefficient estimation and test results in multivariate logistic regression analysis for cognitive impairment. SHS, suboptimal health status; CHD, coronary heart disease.

Table S1 Suboptimal Health Status Questionnaire-25.

Scale	1	2	3	4	5
**Response**	**never or almost never**	**now and then**	**often**	**very often**	**always**
**In the past 3 months how often is it, that you**					
**Fatigue (9 items)**					
1. were exhausted without physical actives significantly increasing	[ ]	[ ]	[ ]	[ ]	[ ]
2. fatigue could not be substantially alleviated by rest	[ ]	[ ]	[ ]	[ ]	[ ]
3. were lethargic in your daily life	[ ]	[ ]	[ ]	[ ]	[ ]
4. suffered from headaches	[ ]	[ ]	[ ]	[ ]	[ ]
5. suffered from dizziness	[ ]	[ ]	[ ]	[ ]	[ ]
6. eyes were aching and tired	[ ]	[ ]	[ ]	[ ]	[ ]
7. muscles or joints felt stiff	[ ]	[ ]	[ ]	[ ]	[ ]
8. have pains in shoulder / neck / waist	[ ]	[ ]	[ ]	[ ]	[ ]
9. have heavy feeling in legs when walking	[ ]	[ ]	[ ]	[ ]	[ ]
**Cardiovascular system (3 items)**					
10. got out of breath while sitting still	[ ]	[ ]	[ ]	[ ]	[ ]
11. suffered from sore throat	[ ]	[ ]	[ ]	[ ]	[ ]
12. were bothered by heart palpitation	[ ]	[ ]	[ ]	[ ]	[ ]
**Digestive system (3 items)**					
13. got poor appetite	[ ]	[ ]	[ ]	[ ]	[ ]

14. suffered from an upset stomach	[ ]	[ ]	[ ]	[ ]	[ ]
15. suffered from indigestion	[ ]	[ ]	[ ]	[ ]	[ ]
**Immune system (3 items)**					
16. got tender fever or cold in-tolerance	[ ]	[ ]	[ ]	[ ]	[ ]
17. were caught with colds in the past 1 year	[ ]	[ ]	[ ]	[ ]	[ ]
18. suffered from sore throat	[ ]	[ ]	[ ]	[ ]	[ ]
**Mental Health (5 items)**					
19. had difficulty in falling asleep	[ ]	[ ]	[ ]	[ ]	[ ]
20. had trouble with waking up during night	[ ]	[ ]	[ ]	[ ]	[ ]
21. had trouble with impairment in short memory	[ ]	[ ]	[ ]	[ ]	[ ]
22. could not respond quickly	[ ]	[ ]	[ ]	[ ]	[ ]
23. had difficulty in concentration	[ ]	[ ]	[ ]	[ ]	[ ]
24. were distracted for no reason	[ ]	[ ]	[ ]	[ ]	[ ]
25. were keyed up or jittery	[ ]	[ ]	[ ]	[ ]	[ ]

Table S2 The Mini-Mental State Examination scores in the healthy and suboptimal healthy status groups.

Dimensions	Levels	Healthy	SHS	*Z*	*P*
Total score	Mean±SD	28.15±2.07	27.56±2.42	3.391	0.001
	Median (P_{25}~P_{75})	29 (27~30)	28 (27~29)		
Orientation	Mean±SD	9.95±0.32	9.93±0.28	1.666	0.096
	Median (P_{25}~P_{75})	10 (10~10)	10 (10~10)		
Registration	Mean±SD	2.96±0.26	2.90±0.46	2.563	0.010
	Median (P_{25}~P_{75})	3 (3~3)	3 (3~3)		
Attention and calculation	Mean±SD	4.45±1.07	4.03±1.49	3.470	0.001
	Median (P_{25}~P_{75})	5 (4~5)	5 (4~5)		
Recall	Mean±SD	2.35±0.87	2.22±0.93	1.670	0.095
	Median (P_{25}~P_{75})	3 (2~3)	2 (2~3)		
Language	Mean±SD	8.45±0.75	8.48±0.63	0.107	0.915
	Median (P_{25}~P_{75})	9 (8~9)	9 (8~9)		

SHS, suboptimal health status; SD, standard deviation.

Table S3 Prevalence rates of cognitive impairment in the subgroups of other demographics.

Subgroups	*n*	Cases	Male cases	Female cases
**Per-capita living space (m^2)**				
≤30 (n, %)	792	40 (5.1)[a]	14 (3.7)[a]	26 (6.3)
30~59 (n, %)	1430	46 (3.2)[a,b]	11 (1.5)[a]	35 (5.0)
≥60 (n, %)	1300	35 (2.7)[b]	9 (1.4)[a]	26 (3.9)
P-value		0.014	0.021	0.211
**N. of family members**				
One (n, %)	138	7 (5.1)	1 (2.0)	6 (6.8)
two ~ four (n, %)	3198	103 (3.2)	29 (1.8)	74 (4.7)
Five or more (n, %)	186	11 (5.9)	4 (4.3)	7 (7.4)
P-value		0.082	0.229	0.331
**Family hygiene**				
Very Good (n, %)	2397	75 (3.1)	21 (1.8)	54 (4.5)
Good (n, %)	1110	46 (4.1)	13 (2.4)	33 (5.8)
Poor (n, %)	16	0 (0)	0 (0)	0 (0)
P-value		0.231	0.587	0.455

Same letters marked in any two subgroups indicate no statistically significant differences; Completely different letters in any two subgroups indicate statistically significant differences.

Table S4 Prevalence rates of cognitive impairment in other behavior-related subgroups.

Behavioral factors	*n*	Cases	Male cases	Female cases
**Raw vegetables**				
None (n, %)	2359	79 (3.3)	20 (1.7)	59 (5.0)
Often (n, %)	1112	40 (3.6)	13 (2.4)	27 (4.8)
Daily (n, %)	53	2 (3.8)	1 (3.4)	1 (4.2)
P-value		0.923	0.550	0.975
**Fried food**				
None (n, %)	3195	108 (3.4)[a]	28 (1.8)[a]	80 (4.9)
Often (n, %)	322	11 (3.4)[a]	4 (2.2)[a]	7 (5.0)
Daily (n, %)	7	2 (28.6)[b]	2 (50.0)[b]	0 (0)
P-value		0.001	<0.001	0.923
**Spicy food**				
None (n, %)	2722	104 (3.8)[a]	27 (2.1)[a]	77 (5.5)
Often (n, %)	760	15 (2.0)[b]	5 (1.2)[a]	10 (2.9)
Daily (n, %)	42	2 (4.8)[a,b]	2 (10.5)[b]	0 (0)
P-value		0.042	0.013	0.076
**Pickled food**				
None (n, %)	3145	101 (3.2)	26 (1.7)[a]	75 (4.7)
Often (n, %)	362	17 (4.7)	6 (3.0)[a]	11 (6.7)
Daily (n, %)	14	2 (14.3)	2 (20.0)[b]	0 (0)
P-value		0.138	<0.001	0.477
**Bacon**				
None (n, %)	3372	117 (3.5)	32 (1.9)[a]	85 (5.0)

Often (n, %)	146	3 (2.1)	1 (1.1)[a]	2 (3.7)
Daily (n, %)	5	1 (20.0)	1 (33.3)[b]	0 (0)
P-value		0.083	<0.001	0.870
**Garlic**				
None (n, %)	2709	96 (3.5)	22 (1.7)	74 (5.1)
Often (n, %)	789	24 (3.0)	11 (2.3)	13 (4.1)
Daily (n, %)	26	1 (3.8)	1 (5.3)	0 (0)
P-value		0.788	0.425	0.637
**Unboiled water**				
None (n, %)	3320	110 (3.3)	29 (1.8)	81 (4.8)
Often (n, %)	148	9 (6.1)	4 (4.6)	5 (8.2)
Daily (n, %)	56	2 (3.6)	1 (3.4)	1 (3.7)
P-value		0.194	0.148	0.464
**Stay up late**				
None (n, %)	2766	102 (3.7)[a]	30 (2.2)	72 (5.2)[a,b]
Often (n, %)	523	8 (1.5)[b]	3 (1.1)	5 (1.9)[b]
Daily (n, %)	234	11 (4.7)[a]	1 (0.9)	10 (8.1)[a]
P-value		0.025	0.371	0.021

The same letters marked in any two subgroups indicate no statistically significant differences; Completely different letters in any two subgroups indicate statistically significant differences.
